# Usefulness of pro-gastrin-releasing peptide as a predictor of the incidence of brain metastasis and effect of prophylactic cranial irradiation in patients with limited-stage small-cell lung cancer

**DOI:** 10.1093/jrr/rrac035

**Published:** 2022-07-02

**Authors:** Kazuhito Ueki, Yukinori Matsuo, Noriko Kishi, Masahiro Yoneyama, Hironori Yoshida, Yuichi Sakamori, Hiroaki Ozasa, Toyohiro Hirai, Takashi Mizowaki

**Affiliations:** Department of Radiation Oncology and Image-Applied Therapy, Graduate School of Medicine, Kyoto University, Kyoto 606-8507, Japan; Department of Radiation Oncology and Image-Applied Therapy, Graduate School of Medicine, Kyoto University, Kyoto 606-8507, Japan; Department of Radiation Oncology and Image-Applied Therapy, Graduate School of Medicine, Kyoto University, Kyoto 606-8507, Japan; Department of Radiation Oncology and Image-Applied Therapy, Graduate School of Medicine, Kyoto University, Kyoto 606-8507, Japan; Department of Respiratory Medicine, Graduate School of Medicine, Kyoto University, Kyoto 606-8507, Japan; Department of Respiratory Medicine, Graduate School of Medicine, Kyoto University, Kyoto 606-8507, Japan; Department of Respiratory Medicine, Graduate School of Medicine, Kyoto University, Kyoto 606-8507, Japan; Department of Respiratory Medicine, Graduate School of Medicine, Kyoto University, Kyoto 606-8507, Japan; Department of Radiation Oncology and Image-Applied Therapy, Graduate School of Medicine, Kyoto University, Kyoto 606-8507, Japan

**Keywords:** limited-stage small-cell lung cancer (LS-SCLC), prophylactic cranial irradiation (PCI), brain metastasis (BM), pro-gastrin-releasing peptide (ProGRP), tumor marker

## Abstract

Prophylactic cranial irradiation (PCI) is recommended for patients with limited-stage small-cell lung cancer (LS-SCLC) who respond well to initial treatment. However, PCI is often omitted because of its potential neurotoxicity in the era of modern diagnostic imaging devices. In the present study, we aimed to investigate the risk factors for brain metastasis (BM) in patients eligible for PCI and who may benefit more from it. Patients with LS-SCLC who responded well to definitive thoracic chemoradiotherapy were included in the present study. Competing risk regression was used to identify factors associated with BM, and the Kaplan–Meier method was used to assess overall survival (OS). Between 2004 and 2017, 62 patients were eligible for PCI and were analyzed. Of these, 38 (61.3%) underwent PCI. Overall, 17 patients (27.4%) developed BM, with a 2-year cumulative incidence of 22.8%. Multivariate analysis (MVA) revealed that pretreatment elevated pro-gastrin-releasing peptide (ProGRP) levels were associated with an increased risk for BM (HR, 7.96, *P* = 0.0091). PCI tended to reduce the risk of BM (HR, 0.33; *P* = 0.051). The use of PCI was associated with improved OS in patients with ProGRP levels > 410 pg/mL (*P* = 0.008), but not in those with ProGRP ≤ 410 pg/mL (*P* = 0.9). Pretreatment ProGRP levels may be useful in predicting the development of BM in patients with LS-SCLC who achieved a good response to initial therapy and to determine which patients should undergo PCI.

## INTRODUCTION

Small-cell lung cancer (SCLC) accounts for approximately 15% of lung cancers [1] and is characterized by its rapid progression and potential for widespread metastasis. Patients with tumors that can be encompassed within an acceptable radiation portal (historically defined as a hemithorax [2]) are classified as having limited disease, which accounts for approximately one-third of all SCLC cases. Thoracic chemoradiation is the standard of care for patients with limited-stage SCLC (LS-SCLC) [3, 4]. However, chemotherapy has limited efficacy in the central nervous system owing to its low penetration of the blood–brain barrier, and approximately 59% to 69% of patients develop brain metastasis (BM) during the course of the disease [4]. This realization has led to the use of prophylactic cranial irradiation **(**PCI) being considered to minimize the incidence of BM and its associated mortality. A meta-analysis of seven randomized trials showed that the use of PCI resulted in an absolute 5.4% improvement in overall survival (OS) at 3 years, establishing PCI as the standard of care for LS-SCLC after patients respond well to thoracic radiotherapy (TRT) [5, 6]. However, PCI is associated with potential neurotoxicity [7, 8], thus, not all eligible patients undergo PCI in clinical practice [9–11]. In addition, the widespread use of magnetic resonance imaging (MRI) and treatments of BM other than whole-brain radiotherapy, such as stereotactic irradiation, has led to debate regarding the role of PCI [12]. However, concerns that omitting PCI may accelerate the development of BM, leading to decreased survival, remain. Given the controversies surrounding PCI, treatment strategies for BM should be optimized by examining those who are at an increased risk for BM and may benefit from PCI. Few reports describing the risk factors of BM in patients with LS-SCLC who achieved a good response to TRT and became eligible for PCI have been written to date. Thus, the aim of this study was to investigate the risk factors for BM in patients eligible for PCI to provide a reference for patients who would benefit from PCI.

## MATERIALS AND METHODS

### Patients

The eligibility criteria for this study were as follows: pathologically confirmed primary SCLC, an Eastern Cooperative Oncology Group performance status of 0–2, received TRT with curative intent, use of concurrent or sequential chemotherapy, and achievement of a complete response (CR) or near CR to TRT according to the Response Evaluation Criteria in Solid Tumors (RECIST), version 1.1. Patients who were lost to follow-up within 6 months of TRT were excluded from the study. Limited disease was defined as a tumor that was confined to one hemithorax with or without mediastinal lymph nodes and/or supraclavicular lymph nodes, provided that all volumes could be included in the same radiotherapy field. Clinical staging was performed in accordance with the 8^th^ Union for International Cancer Control Tumor, Node, Metastasis (TNM) staging system using computed tomography (CT) of the thorax and abdomen and CT or MRI of the brain. ^18^F-fluorodeoxyglucose positron emission tomography/computed tomography (FDG-PET) was also performed.

Plasma pro-gastrin-releasing peptide (ProGRP) levels and serum neuron-specific enolase (NSE) levels were measured at baseline and after the initial treatment was completed as appropriate. The ProGRP concentration was measured using a CLIA kit (Abbott Japan LLC, Tokyo, Japan), whereas the NSE concentration was measured using an ECLIA kit (Roche Inc., Tokyo, Japan). The upper normal limits for ProGRP and NSE levels were 81.0 pg/ml and 12.0 ng/ml, respectively. The present study was approved by the relevant Institutional Review Board and the requirement for written informed consent was waived owing to the retrospective study design. This research was conducted in accordance with the principles of Declaration of Helsinki.

### Treatment and follow-up

TRT was delivered with accelerated hyperfractionated radiotherapy (twice daily, 45 Gy in 30 fractions over 3 weeks) or conventional-fractionated radiotherapy (once daily, 50 Gy in 25 fractions over 5 weeks). All patients received either concurrent or sequential platinum-based chemotherapy consisting primarily of etoposide combined with cisplatin or carboplatin. After the initial therapy was completed, the treatment response was evaluated using thorax/abdominal CT and CT or MRI of the brain to ensure eligibility for PCI. FDG-PET was performed optionally. PCI was indicated for patients who attained a CR or near CR based on RECIST version 1.1. PCI was administered once daily at a total dose of 25 Gy in 10 fractions over 2 weeks. For standard workup for recurrence, patients underwent thorax/abdominal CT every 3–6 months after the initial treatment. Brain MRI was also performed at intervals of 3–6 months. However, not all patients underwent regular brain MRI, and head CT was substituted in some cases or MRI was performed when BM was suspected.

### Statistics

Baseline characteristics of patients between groups were compared using the chi-square test or Fisher’s exact test for categorical variables and the Mann–Whitney U-test for continuous variables. The time of clinical events, such as recurrence or death, was defined as the date from the date of chemotherapy or radiotherapy to the date of recurrence, death, or the last follow-up. The cumulative incidence of BM or extracranial metastasis (ECM) was estimated using the cumulative incidence function, accounting for deaths as competing risk, and the groups were compared using Gray’s test. Variables found to be significant in the univariate analysis (UVA) and variables that we decided should be adjusted within the model from a clinical perspective were included in the Fine-Gray model during multivariate analysis (MVA). OS was estimated using the Kaplan–Meier method, and the groups were compared using a univariate log-rank test. Predictive performance of tumor markers was assessed by plotting receiver operating characteristic (ROC) curves and estimating the area under the ROC curves (AUC). All tests were two-sided, and statistical significance was defined as a *P*-value of < 0.05. All statistical analyses were performed using R software (version 3.6.3, www.r-project.org).

## RESULTS

### Eligible patients

**Fig. 1:**
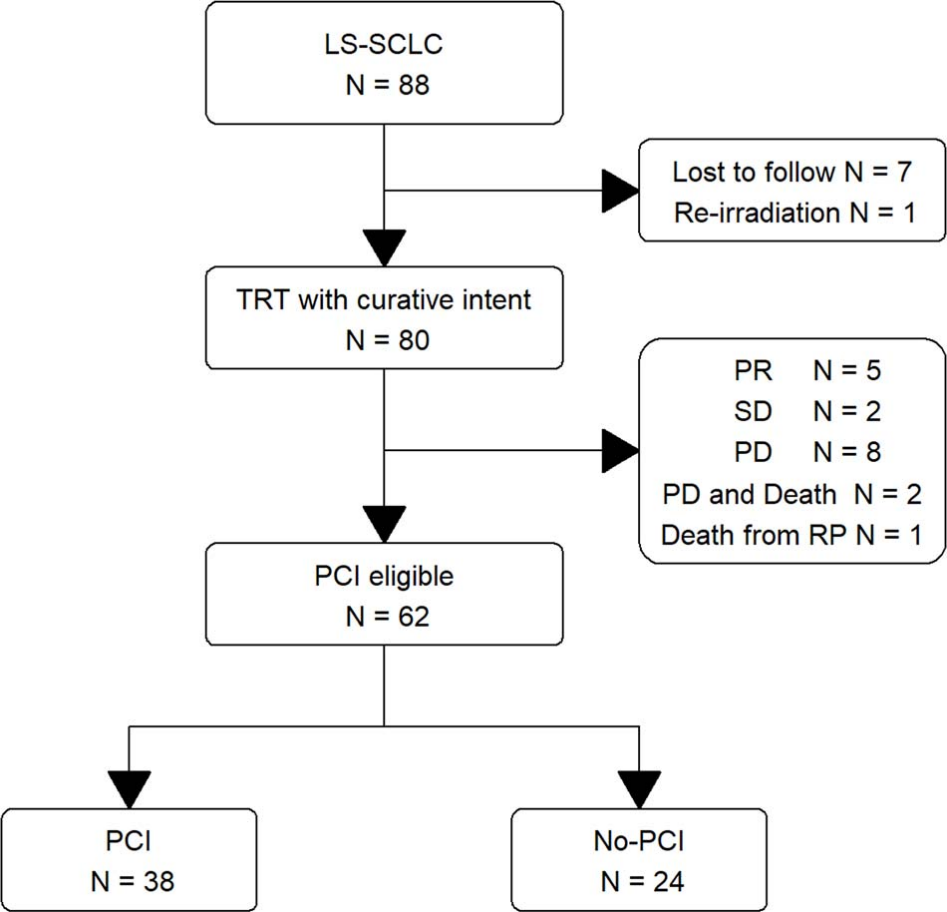
Flow chart of patient selection. Abbreviations: LS-SCLC, limited-stage small cell lung cancer; TRT, thoracic radiotherapy; PCI, prophylactic cranial irradiation; PR, partial response; CR, complete response; PD, progressive disease; RP, radiation pneumonitis.

In total, 88 patients were diagnosed with LS-SCLC at our institution between 2004 and 2017, 80 of whom underwent TRT for curative purposes (Fig. 1). After excluding patients who did not meet the selection criteria, 62 patients who attained CR (*n* = 32) or near CR (*n* = 30) who were eligible for PCI were analyzed. Of these, 38 underwent PCI (PCI group). The remaining 24 (non-PCI group) were eligible for PCI but did not undergo it for the following reasons: high age or deterioration of performance status (*n* = 6), patient refusal (*n* = 7), comorbidity (pyothorax, colon cancer, lung cancer) (*n* = 3), radiation pneumonitis (*n* = 5) or unknown (n = 3).

### Patient characteristics

**Fig. 2:**
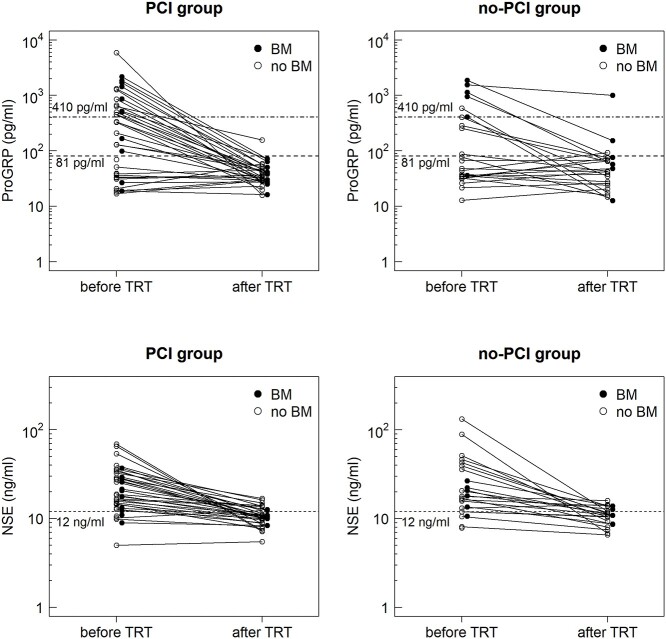
Tumor marker levels before and after TRT in the PCI group (*n* = 38) and non-PCI group (*n* = 24). The horizontal dashed lines indicate the upper normal limits. The horizontal dotdash lines in the graphs for ProGRP indicate the ProGRP level of 410 pg/ml. Filled and open circles represent patients who developed BM and those who did not, respectively.

Among the baseline characteristics, age differed significantly between the PCI and non-PCI groups (Table 1). Brain MRI was performed for 85% (53/62) of patients as a part of the initial staging, among whom contrast media was used for 94% (50/53). Every patient in the PCI group underwent brain imaging prior to undergoing PCI (73.7% for MRI), whereas 66.7% of the patients in the non-PCI group did not undergo brain imaging after TRT (Table 1). The percentage of the use of contrast media for MRI after TRT was 97% (32/33). The positivity rates of tumor markers before TRT were 54.8% and 82.3% for ProGRP and NSE, respectively (Table 1 and Fig. 2). Among the patients with elevated tumor marker levels before TRT, normalization occurred after TRT in 79.4% for ProGRP and 60% for NSE (Fig. 2). ProGRP and NSE were not measured after treatment in two and four patients with normal baseline tumor marker levels, respectively. NSE was not measured after treatment in one patient with elevated NSE levels at baseline.

**Table 1:** Patient characteristics

characteristics	Non-PCI (n = 24)		PCI (n = 38)		*P*-value
Age (median [IQR])	72	[69, 75]	65.5	[59, 69]	< 0.001
Sex (%)					0.629
M	18	(75.0)	25	(65.8)	
F	6	(25.0)	13	(34.2)	
Performance status (%)					0.617
0	9	(37.5)	18	(47.4)	
1–2	15	(62.5)	20	(52.6)	
Stage (%)					0.294
I	2	(8.3)	1	(2.6)	
II	4	(16.7)	11	(28.9)	
III	18	(75.0)	26	(68.4)	
Pre-TRT ProGRP (%)					0.163
Normal	14	(58.3)	14	(36.8)	
Elevated	10	(41.7)	24	(63.2)	
Pre-TRT NSE (%)					0.736
Normal	5	(20.8)	6	(15.8)	
Elevated	19	(79.2)	32	(84.2)	
Radiotherapy (%)					0.222
AHF	17	(70.8)	32	(84.2)	
CF	7	(29.2)	6	(15.8)	
Chemotherapy (%)					0.366
Concurrent	21	(87.5)	36	(94.7)	
Sequential	3	(12.5)	2	(5.3)	
Pre-TRT brain image (%)					0.077
MRI	18	(75.0)	35	(92.1)	
CT	6	(25.0)	3	(7.9)	
Post-TRT brain image (%)					< 0.001
MRI	5	(20.8)	28	(73.7)	
CT	3	(12.5)	10	(26.3)	
None	16	(66.7)	0	(0.0)	

Abbreviations: PCI, prophylactic cranial irradiation; IQR interquartile range; TRT, thoracic radiotherapy; CF, conventional-fractionated; AHF, accelerated hyperfractionated; CT, computed tomography; MRI, magnetic resonance imaging.

### Risk Factors for BM

**Fig. 3:**
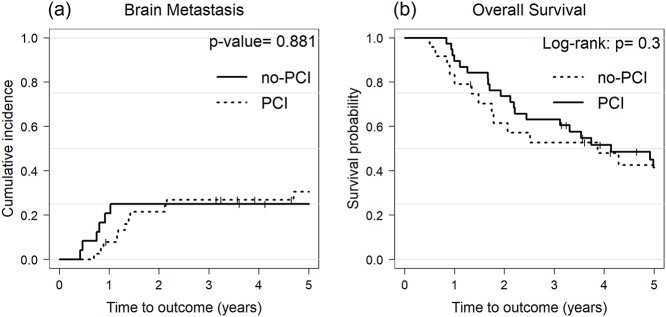
Cumulative incidence of BM and OS stratified according to PCI.

**Fig. 4:**
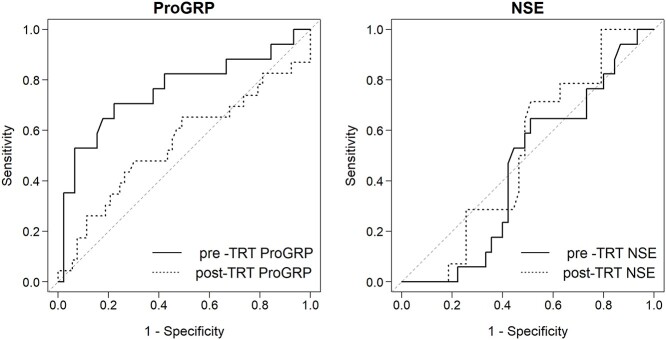
ROCs of tumor markers for predicting BM. Abbreviations: ProGRP, pro-gastrin-releasing peptide; NSE, neuron-specific enolase.

**Fig. 5:**
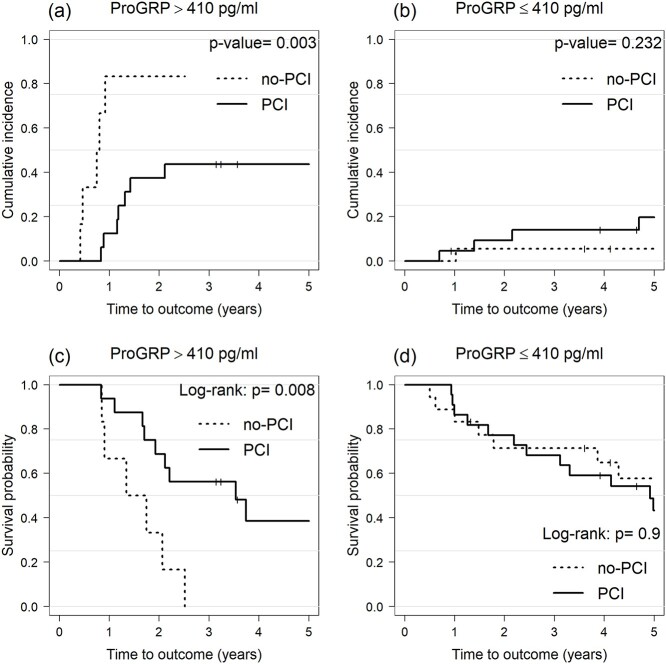
Comparison between the cumulative incidence of BM (a, b) and OS (c, d) in the PCI and non-PCI groups stratified according to ProGRP levels (> 410 pg/ml vs ≤ 410 pg/ml).

Overall, 17 patients (17/62, 27.4%) developed BM (2-year cumulative incidence, 22.8%). The UVA showed that ProGRP before TRT (pre-TRT) was the only factor significantly associated with BM incidence (normal vs elevated, 2-year cumulative incidence: 11% vs 32.4%; *P* = 0.01, Table 2). Although PCI was not significantly associated with the incidence of BM in UVA (Table 2 and Fig. 3a), given that ProGRP levels were associated with the incidence of BM, we hypothesized that the effect of PCI on reducing BM may vary based on ProGRP levels. To evaluate this speculation, we included PCI in the multivariate model in addition to ProGRP levels (normal vs elevated). The MVA demonstrated that the use of PCI trended toward a lower BM incidence (HR, 0.33; 95% confidence interval [CI], 0.11–1.00; *P* = 0.051), and elevated ProGRP levels remained a significant predictive factor of BM (HR, 7.96; 95% CI, 1.68–37.80; *P* = 0.0091). In the ROC curves predicting BM, the AUC value for ProGRP before TRT was the largest compared to those for post-TRT ProGRP, pre- and post-TRT NSE, with AUC values of 0.76 (95% CI, 0.60–0.91), 0.55 (95% CI, 0.39–0.70), 0.45 (95% CI, 0.30–0.60), and 0.51 (95% CI, 0.36–0.67), respectively (Fig. 4). The cutoff value of 410 pg/ml for pre-TRT ProGRP had a sensitivity of 71%, specificity of 78% in predicting BM. Patients with pre-TRT ProGRP levels > 410 pg/ml (35% of the entire cohort) were at significantly higher risk for BM compared to those with pre-TRT ProGRP levels ≤ 410 pg/ml (≤ 410 pg/ml vs > 410 pg/ml, 2-year cumulative incidence, 7.6% vs 50%; *P* < 0.001). Stratified cumulative incidence curves showed that the use of PCI was significantly associated with a lower incidence of BM among patients with pre-TRT ProGRP levels > 410 pg/ml (2-year cumulative incidence for PCI group vs non-PCI group, 37.5% vs 83.3%; *P* = 0.003, Fig. 5a). Conversely, the incidence of BM in patients with pre-TRT ProGRP levels ≤ 410 pg/ml was comparable between the PCI and non-PCI groups (2-year cumulative incidence for the PCI group vs non-PCI group, 9.3% vs 5.6%; *P* = 0.232, Fig. 5b).

**Table 2:** UVA for BM and ECM

	BM		ECM	
	2y rate (%)	*P*-value	2y rate (%)	*P*-value
Age		0.606		0.403
≥ 70	20		28	
< 70	24.7		35.1	
Sex		0.871		0.423
Male	21.1		37.2	
Female	26.3		21.1	
PS		0.657		0.362
0	25.9		25.9	
1–2	20.4		37.1	
Stage		0.972		0.828
I–II	27.8		33.3	
III	20.6		31.8	
Radiotherapy		0.668		0.285
AHF	24.8		28.6	
CF	15.4		46.2	
Chemotherapy		0.475		0.452
Concurrent	21.3		33.3	
Sequential	40		20	
pre-TRT ProGRP		0.01		0.156
Normal	11		21.4	
Elevated	32.4		41.2	
pre-TRT NSE		0.927		0.18
Normal	18.2		45.5	
Elevated	23.8		29.4	
post-TRT ProGRP		0.285		0.131
Normal	21.7		32.1	
Elevated	50.0		50.0	
post-TRT NSE		0.883		0.111
Normal	23.1		33.3	
Elevated	22.6		16.7	
Response		0.291		0.51
CR	15.9		31.2	
near CR	30.0		33.3	
PCI		0.881		0.802
Yes	21.5		31.6	
No	25		33.3	

Abbreviations: BM, brain metastasis; ECM, extracranial metastasis; PS, performance status; CH conventional-fractionated; AHF, accelerated hyperfractionated; TRT, thoracic radiotherapy; CR, complete remission; PCI, prophylactic cranial irradiation.

### Risk Factors for ECM

Overall, 17 patients (30/62, 48%) developed ECM. None of the factors were significantly associated with the incidence of ECM in the UVA (Table 2). The difference in the incidence of ECM in the dichotomization of the cohort by applying the ProGRP level of 410 pg/ml was not significant (*P* = 0.58).

### Impact of PCI on OS according to ProGRP levels

Overall, 40 patients (64.5%) died during the follow-up period and the median OS was 2.8 years. The Kaplan–Meier curves for OS according to the use of PCI did not differ significantly (*P* = 0.3, Fig. 3b). As the use of PCI was associated with a lower incidence of BM, especially in patients with higher pre-TRT ProGRP levels (Fig. 5a), we compared the OS in the PCI group with that in the non-PCI group and stratified it according to the pre-TRT ProGRP levels (≤ 410 pg/ml vs > 410 pg/ml). PCI use was associated with a superior OS (2-year rate in the PCI group compared to the non-PCI group, 68.8% vs 33.3%, *P* = 0.008, Fig. 5c) among patients with pre-TRT ProGRP levels of > 410 pg/ml. In contrast, OS in patients with pre-TRT ProGRP levels ≤ 410 pg/ml was comparable between the PCI and non-PCI groups (2-year rate for PCI group vs non-PCI group, 77.3% vs 71.4%, *P* = 0.9, Fig. 5d).

## DISCUSSION

The rationale for PCI is usually that some patients attain excellent systemic control of the disease, whereas those who experience a central nervous system relapse tend to die.^10^ Therefore, a more precise prediction of the risk of BM would be of significant clinical utility since low-risk patients could avoid the potential toxicity of PCI with little or no detriment to their survival, and higher-risk patients will have more assurance that PCI improves their prognosis, justifying the treatment risks. The risk factors for BM in patients eligible for PCI are poorly defined to our knowledge. Several studies have examined the risk factors for BM in patients with LS-SCLC, including both eligible and ineligible patients for PCI. Wu *et al.* reviewed 283 patients with stage I–IIIB SCLC and determined that the TNM stage predicted the likelihood of distant metastasis, BM and OS. PCI was not significantly associated with a lower risk of BM, but with improved OS in their study [13].^13^ A report by Farooqi *et al.* revealed that PCI reduced the risk of BM and death in the entire cohort [14].^14^ However, PCI did not improve OS among patients aged ≥70 years with ≥5 cm tumors. Both studies included several patients in an observational study of LS-SCLC and provided important insights into the risk of BM in patients with LS-SCLC. However, PCI is indicated for patients with a good response to initial therapy as recognized by Wu *et al.,* which may introduce selection bias into the analysis. In other words, the non-PCI group in their studies included both those who responded well to initial treatment but did not undergo PCI and those who did not have a good response and were therefore ineligible for PCI. We believe that including the latter group of patients in the survival analysis may result in inappropriate adjustment in the multivariate model, as the effect of PCI may be assessed even in patients who are ineligible for PCI. Therefore, the present study investigated the risk factors of BM and the effect of PCI in eligible patients to answer the clinical question of who among the eligible patients was more likely to develop BM and benefit from PCI. Our results showed that pre-TRT ProGRP levels were a risk factor for the development of BM in both UVA and MVA. Among patients with ProGRP levels > 410 pg/ml, the use of PCI was significantly associated with a lower incidence of BM and improved OS. Our results suggest that pre-TRT ProGRP levels before TRT may be useful in predicting BM in patients eligible for PCI and in considering whether an eligible patient should undergo PCI.

ProGRP is reportedly a useful tumor marker for diagnosis, monitoring therapeutic response, and detecting recurrence in patients with SCLC [15, 16]. Several other studies have also reported the prognostic value of ProGRP. Wojcik *et al.* and Nisman *et al.* demonstrated that a ProGRP level of > 410 ng/l and > 800 ng/l were associated with reduced OS in patients with SCLC, respectively [17, 18]. However, few studies have reported an association between ProGRP levels and the incidence of BM in patients with LS-SCLC to date. Yonemori *et al.* investigated the risk factors for BM in patients with LS-SCLC who underwent PCI [19]. In their cohort, 50% of patients had elevated serum ProGRP levels (> 46 pg/ml) before PCI, and elevated ProGRP levels before PCI were significant predictive and prognostic factors for first failure events due to BM, overall BM incidence, and survival. Based on their results, they speculated that elevated ProGRP levels before PCI might reflect the existence of residual viable tumor cells after induction treatments, even if CR or good PR is achieved on imaging studies. However, only 6.5% (4/62) of the entire cohort had ProGRP levels above the normal cut-off values after TRT in the present study (Fig. 2). Therefore, we could not evaluate the impact of ProGRP levels before PCI because of the small number of patients. However, unlike the study by Yonemori *et al.*, the results of our study suggest that high ProGRP levels at baseline reflect the presence of occult BMs that were not detected on baseline imaging or the potential aggressiveness of tumors.

ProGRP is a biologically active protein that stimulates tumor cell proliferation [20, 21]. Gastrin-releasing peptide, which is thought to be derived mostly from ProGRP [22, 23], has also been reported to function as an autocrine growth factor for SCLC [20, 24]. Thus, the growth-stimulating effects of ProGRP may be responsible for aggressive tumor behavior and poor prognosis. This biological mechanism of ProGRP is consistent with the results of a study that reported that ProGRP was highly elevated in patients with metastasis [25]. In the present study, high ProGRP levels were significantly associated with an increased incidence of BM, but not with ECM. One reasonable explanation for this is that TRT contributed to the inhibition of repopulation and dissemination by rapid destruction of thoracic tumors, and concomitant chemotherapy was sufficiently effective to control micrometastases outside the thoracic irradiation volume. Therefore, we believe that ProGRP is not a tumor marker that is particularly elevated in patients with BM, but it is useful for predicting tumor growth in the brain, a sanctuary to which chemotherapy is difficult to transfer among the select patients who responded well to initial therapy. Thus, it may be important to consider the indications for PCI based not only on the response to TRT but also on the assessment of tumor aggression. There are few ways to assess tumor aggressiveness in SCLC. Quantitative measures of biological invasiveness, such as FDG uptake, are generally better indicators of survival and disease progression [26]; However, it is unclear whether FDG-PET has prognostic value in patients with LS-SCLC [27]. Measuring tumor marker concentrations is less expensive and easier than measurement using imaging modalities.

Pre-TRT ProGRP showed better predictive ability for BM than pre- and post-TRT NSE. Although NSE has become a widely used laboratory assay of patients with SCLC [28], the diagnostic and prognostic sensitivity and specificity of NSE have been found to be lower than those of ProGRP, especially in early stages of SCLC [15, 17, 18, 29]. One possible reason for this difference is that NSE depends on tumor burden, while ProGRP can reflect tumor activity of SCLC and reach high levels already in its limited disease [16]. This may have resulted in NSE being inferior to ProGRP in detecting microscopic BMs.

In the present study, although the use of PCI tended to result in a reduced incidence of BM in the MVA, it was not associated with improved OS in the entire cohort. We speculate that the lack of a clear impact of PCI on OS may be related to the fact that our BM rate was lower than that reported in previous studies that provided a rationale for recommending PCI for patients with LS-SCLC [5, 6]. As pointed out by several authors, many landmark studies that provided evidence of PCI for LS-SCLC were performed in the era before MRI was routinely used for staging [10, 30, 31]. In addition, a fraction of patients included in the meta-analysis did not have CT scans or had extensive disease [6]. The number of patients classified as having extensive disease has increased in the era of MRI as patients are frequently upstaged based on the detection of asymptomatic disease [31], which would reduce the incidence of BM in patients with LS-SCLC compared to the pre-MRI era. Pezzi *et al.* evaluated the usefulness of PCI in patients with LS-SCLC, all of whom underwent at least baseline MRI and restaging brain MRI and/or head CT [30]. They reported a 20.4% incidence of BM in the non-PCI group, which was comparable to our study in which MRI was a standard method for staging patients with SCLC, with 85.5% undergoing baseline MRI. Furthermore, similar to our study, PCI was not associated with an OS benefit in their study. Although PCI was not associated with improved OS in the entire cohort, the OS was significantly higher in the PCI group among patients with ProGRP levels > 410 pg/mL. Thus, our results do not completely deny the benefit of PCI in the MRI era but suggest that PCI may be effective in a select few patients who are expected to be at an increased risk for BM.

Our study has limitations inherent in its retrospective nature, and involved few patients at a single institution. We restricted our cohort to patients eligible for PCI to eliminate selection bias because PCI is generally indicated in patients with good performance status and response to initial therapy. However, the non-PCI group included patients whose performance status worsened or who experienced a decline in pulmonary function during TRT. Due to the limited data, insufficient adjustments for such confounding factors may have been made to address the heterogeneity between the PCI and non-PCI groups. In addition, not all patients underwent contrast-enhanced (CE) MRI. The use of non-CE MRI makes it difficult to detect a very small metastasis, which may have affected the incidence of BM in this study. Furthermore, our study also included patients treated during a relatively long timeframe during which standard treatment may have changed. Incorporating immunotherapy with platinum-based chemotherapy has recently benefited patients with SCLC [32–34]. Immunotherapy is reportedly effective in treating BM in selected patients with non-small cell lung cancer [35]. The validity of using ProGRP values to select patients for PCI in the immunotherapy era must be examined in future studies.

## CONCLUSION

Pretreatment ProGRP levels were accurate predictors for the incidence of BM in patients with LS-SCLC who achieved a good response to initial therapy. Patients with elevated ProGRP levels (> 410 pg/mL) exhibited improved survival following PCI, indicating that pretreatment with ProGRP may factor in determining who should undergo PCI.
