# Correction to: Usefulness of pro-gastrin-releasing peptide as a predictor of the incidence of brain metastasis and effect of prophylactic cranial irradiation in patients with limited-stage small-cell lung cancer

**DOI:** 10.1093/jrr/rrac070

**Published:** 2022-09-19

This is a correction to: Kazuihto Ueki, Yukinori Matsuo, Noriko Kishi, Masahiro Yoneyama, Hironori Yoshida, Yuichi Sakamori, Hiroaki Ozasa, Toyohiro Hirai, Takashi Mizowaki, Usefulness of pro-gastrin-releasing peptide as a predictor of the incidence of brain metastasis and effect of prophylactic cranial irradiation in patients with limited-stage small-cell lung cancer, *Journal of Radiation Research*, Volume 63, Issue 4, July 2022, Pages 636–645, https://doi.org/10.1093/jrr/rrac035

In the originally published version of this manuscript, there was an error in the author list. The following author's name should read as “Kazuhito Ueki” instead of “Kazuihto Ueki”.

This error has been corrected online.

